# Ambiguity in Social Network Data for Presence, Sensitive-Attribute, Degree and Relationship Privacy Protection

**DOI:** 10.1371/journal.pone.0130693

**Published:** 2015-06-25

**Authors:** Mehri Rajaei, Mostafa S. Haghjoo, Eynollah Khanjari Miyaneh

**Affiliations:** 1 Department of Computer Engineering, Iran University of Science and Technology, Tehran, Iran; 2 PayameNoor University, Kish International branch, Kish, Iran; Mathematical Institute, HUNGARY

## Abstract

Maintaining privacy in network data publishing is a major challenge. This is because known characteristics of individuals can be used to extract new information about them. Recently, researchers have developed privacy methods based on *k*-anonymity and *l*-diversity to prevent re-identification or sensitive label disclosure through certain structural information. However, most of these studies have considered only structural information and have been developed for undirected networks. Furthermore, most existing approaches rely on generalization and node clustering so may entail significant information loss as all properties of all members of each group are generalized to the same value. In this paper, we introduce a framework for protecting sensitive attribute, degree (the number of connected entities), and relationships, as well as the presence of individuals in directed social network data whose nodes contain attributes. First, we define a privacy model that specifies privacy requirements for the above private information. Then, we introduce the technique of Ambiguity in Social Network data (ASN) based on anatomy, which specifies how to publish social network data. To employ ASN, individuals are partitioned into groups. Then, ASN publishes exact values of properties of individuals of each group with common group ID in several tables. The lossy join of those tables based on group ID injects uncertainty to reconstruct the original network. We also show how to measure different privacy requirements in ASN. Simulation results on real and synthetic datasets demonstrate that our framework, which protects from four types of private information disclosure, preserves data utility in tabular, topological and spectrum aspects of networks at a satisfactory level.

## Introduction

**Fig 1:**
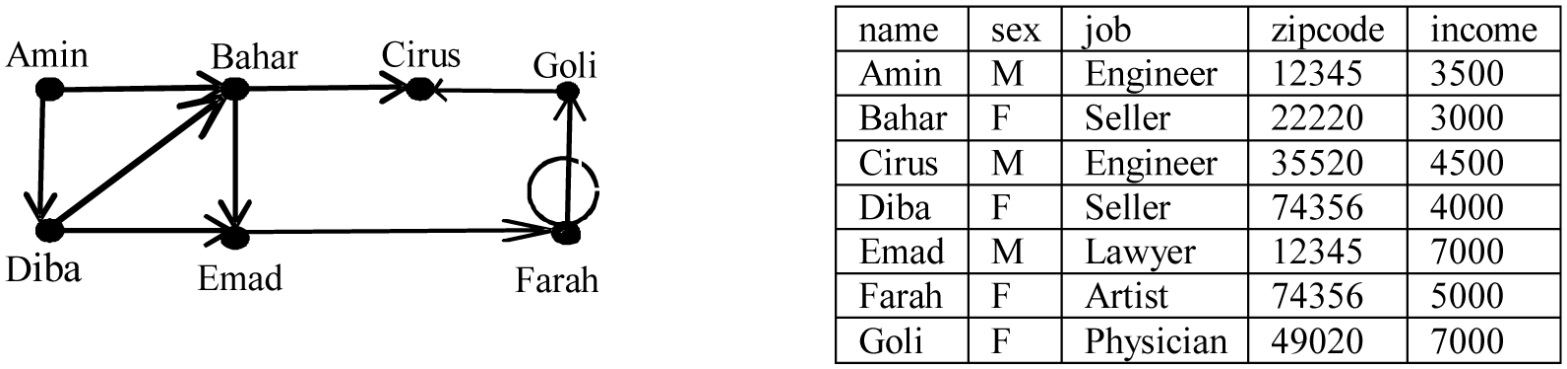
An example of money transformation network.

Nowadays, huge amounts of data are collected by online and offline social networks such as LinkedIn, friendship networks, telephone call networks, academic co-authorship networks, disease infection networks and such like. In recent years, data miners have shown great interest in analysing social network data [1,2]. Such analysis offers rich opportunities to extract key social patterns relating for example to disease spread, influence of publications, and community growth, which could be of interest to politicians, sociologists, economists, commercial companies, lawyers, and so on. But social network data contain sensitive and private information about individuals, such as their relationships with VIPs or organizations in the society. Protecting user privacy when publishing social network data is an important challenge that goes beyond removing identifying attributes of users. The danger is that an *adversary* could use *background knowledge* about the context of an individual’s network for *re-identification*, or identify additional properties about an individual. There are well-known examples of released datasets that have caused a breach of privacy for individuals [3]. The focus of this paper is directed to preserving individual privacy for a directed graph model where each vertex in the graph is associated with non-sensitive and sensitive attributes. Fig 1 shows an example of such a graph.

Several types of privacy disclosure have been introduced in current approaches: (a) content disclosure, which is disclosure of attributes values associated to each individual, such as age, income, etc.; (b) identity disclosure (re-identification), which refers to re-identifying of the node of a target individual by an adversary from background knowledge; (c) degree disclosure, which is disclosure of the degree value of each individual; (d) relationship disclosure, which is the inferring by an adversary of the high probability of the existence of a relationship between two individuals. Most current approaches [4,5,6,7,8,9] satisfy *k*-anonymity [10] to protect against identity disclosure. Their goals are to establish a social network which always has at least *k* candidate individuals in different attack scenarios. Preventing identity disclosure does not guarantee avoidance of other kinds of private-information disclosure [9,11,12,13,14,15,16]. This paper focuses on prevention of disclosure of information related to presence, sensitive attribute, degree and relationships. Presence disclosure [17] is identification of the existence of individuals in published data if an adversary does not know about his or her membership.

Existing approaches in social network data publications consider different scenarios for an adversary’s background knowledge, then prevent disclosure of specific private information based on the specified background knowledge. For example, the method presented in [5] prevents re-identification when an adversary knows the degree of individuals in a network. In this paper, an adversary may know about quasi-identifiers, degree and sensitive attribute of victims. As in real-world applications, this paper considers properties, such as degree and sensitive attribute, as both background knowledge of an adversary and private information. This means that, if the adversary does not already know about that characteristic of a victim, he/she will not discover its value after accessing released data.

### Motivation

Most research approaches treat social networks as undirected graph models, but some real-world social networks are directed graphs, such as email networks, financial transaction networks, disease transmission networks, and ResearchGate. The study in [18] shows that attacks become easier to carry out if the released graph data is directed. In this paper, we consider direction for relationships between individuals on social networks.

Some other studies have been done [12,19] for directed social network publication, however these researches do not consider attributes for individuals. Like [16,20], in the method presented in this paper, each node has labels which may be used by an adversary to identify targets; each node also has sensitive attributes that should be protected as in [6,15,14]. In other words, our network model is a directed graph that involves both structural and tabular data.

The approaches presented in [12,19] only prevent link disclosure by replacing the destination of each relationship with a random node with specified probability. In this paper, we consider four types of disclosure: presence disclosure, sensitive attribute disclosure, degree disclosure and relationship disclosure.

Generalization [21] is a popular methodology to realize *k*-anonymity in relational data. Some studies [6,4,20,16] use generalization for social network anonymization. Specifically, the network is partitioned into groups, and values of attributes of nodes in the same group are replaced by identical generalized values. The structural information of group members is summarized into one super-node. Although this can protect both presence privacy and association privacy, generalization often results in considerable information loss. To address this defect, permutation-based techniques such as anatomy [22], *k*-permutation [23] are presented. In this paper, we use an anatomization technique, which achieves better accuracy of aggregate queries [17] by publishing exact values of attributes and degrees.

### Contributions and Organization

Given all the above considerations, this paper makes the following contributions:

1. We formalize privacy requirements for presence privacy, sensitive attribute privacy, degree privacy, and relationship privacy as a privacy model called (α,β,γ,δ)-SNP for graph and network data. α,β,γ and δ are set by data publisher and specify the maximum probability of disclosure of each individuals private information. These parameters represent a trade-off between privacy and data utility. A more limited privacy constraint leads to decreased data utility.
2. We introduce an anonymization technique *ASN* (Ambiguity Social Network) that describes how to publish directed social network data in order to satisfy (α,β,γ,δ)-SNP privacy requirements. To use ASN, all nodes of a graph should be partitioned into separate groups. Then ASN publishes exact value of quasi-identifiers, sensitive label, degree and successors of members of each group in separate tables with common group ID. Group ID is used to join these tables. This is a lossy join and generates false individuals and relationships. This issue brings uncertainty to reconstruct original graph from published data. We show how to measure probability of disclosure of presence, sensitive attribute, degree and relationship for published data based on ASN.
3. To evaluate data utility of our framework, we develop a simple greedy algorithm for partitioning individuals of a social network into groups for ASN, such that each group satisfies privacy requirements. We analyze the resistance of our framework against an adversary’s background knowledge and evaluate data utility preservation through experiments on both real and synthetic datasets.

The rest of the paper is arranged as follows: section 2 reviews the related work. Section 3 defines the problem. Section 4 presents the ASN approach and section 5 provides methods for measuring privacy requirements for published data based on ASN. Section 6 analyzes defense against different types of background knowledge. Section 7 presents a partitioning algorithm. Finally, we evaluate the utility of our framework in section 8, and conclude in section 9.

## Related Work

Current approaches to anonymization methods on simple network data are categorized in [24] as follows:

1. *Network generalization* clusters nodes and edges into groups and anonymizes a subgraph into a super-node. Details about individuals are hidden. Most methods in this category add nodes to super nodes until each super node contains at least k nodes (these methods often use k-anonymity models). Methods [20,25] prevent disclosure of sensitive attributes and their participation in interaction in a bipartite graph. In [6], nodes and quasi-identifier attributes of nodes are generalized to prevent re-identification. In [4], nodes and edges are clustered to prevent re-identification against structural attacks.
2. *Directed alteration* modifies the graph structure via a sequence of edge deletions and additions such that each node in the modified graph is indistinguishable from at least *k-1* other nodes in terms of some types of structural pattern. The privacy model of these methods is also *k*-anonymity. The study presented in [5] sought to add a minimal set of edges to prevent degree attack. In [16,26], to prevent neighbourhood attack, edges are added until the neighbourhood subgraph of each node is similar to at least k-1 in other nodes. Then, the label values of all nodes of each group are replaced by one generalization value. In [9,7], to prevent re-identification against different structural queries, edges are added to the graph until there are at least k-1 distinct isomorphic subgraphs for each subgraph of the released network. In particular [9] prevents link disclosure. The approaches presented in [8,15] protect against any structural attacks by adding fake nodes and edges. The study presented in [14] splits nodes into multiple substitutes to achieve the desired privacy requirements. All these methods protect re-identification against specified background knowledge. Some such methods [16,15,14] also protect sensitive label and community identity disclosure. These algorithms use dynamic programming and greedy techniques to apply minimum changes in order to preserve the graph structure as much as possible. In all these methods, more protection causes more information loss.
3. *Edge randomization* modifies the graph structure by randomly adding/deleting or switching edges. It protects against re-identification in a probabilistic manner [27,28,29,30,31,32]. In [19,12] new edge-randomization methods are introduced for directed graphs to protect link disclosure. Only the destination of each edge is replaced with one randomly chosen node from subsets of nodes (close to the source node of an edge). In this way, the out degrees of nodes in published data remain unchanged.

Recently, another scenario was proposed for private analysis of social-network data. Here, instead of publishing data, query results are released that satisfy differential-privacy (DP) [33,34]. DP is a privacy standard developed for use on tabular data, which guarantees privacy without making assumptions about an attacker’s background knowledge. DP adds randomized noise into the query result to hide the impact of adding or removing one person’s data from the dataset.

Two problems arise in extending DP for social-network data: (I) Determination of data belonging to each individual is unclear. Two adaptations are proposed to overcome this; edge privacy [35] and node privacy [36]; node privacy provides a high level of privacy. (II) Social network metrics are highly sensitive to small changes in network structure. It is likely that this problem will entail a lot of information loss, because random added noise is very high in some queries. In addition, producing results is not always feasible in some queries because the amount of noise is dependent on the number of nodes in the network [37].

Although DP provides strong privacy against each kind of background knowledge, it has some drawbacks. The study presented in [38] illustrates one example of how DP can violate the privacy of individuals in social networks. In addition, for each kind of analysis, an algorithm should be developed to compute results based on DP. The studies presented in [35,36,37,39,40] present some algorithms for minimal spanning tree, number of specific subgraphs, and degree distribution. As query-answering is done online, generating results to satisfy DP can be time-consuming in some analyses. The amount of noise is very high for some private analyses, and this incurs a lot of information loss. Finally, we note that privacy-preserving social network publishing has some advantages over private analysis of social networks. Data publishers firstly anonymize data one time offline and then publish, but this enables a data miner to extract a result for each query.

## Problem Statement

In this section we specify the social-network model, privacy requirements and adversary background knowledge. In some real-world social-network applications, relationships between entities are *directed* with the possibility of loops (same source and destination). We model these networks as directed graphs so heads and tails of each directed edge have a different role. For example, in an email communication network each email address is represented by a node and each email is represented by a directed edge from sender to receiver. We allow only binary relationships in our network model (only two individuals are involved in each relationship). Moreover, we assume that all relationships have the same properties, so directed edges do not have labels to specify them (because we do not protect against relationship type in this paper). We let entities have three types of labels to describe their properties. One of these labels is *sensitive*, i.e., that which has to be protected. For example, in a product distribution network, the income of a company may be a sensitive attribute.

The formal definition of our network structure is as follows:

**Definition 1:** *Social-network data* is a directed graph *N* = (*V*, *E*)in which *V* is the set of individuals (|*V*| = *n*) and *E* is the set of relationships on *V* (|*E*| = *m*). Elements of *E* are directed edges or arcs. Repeated arcs (*i*, *j*) are not allowed, and multiple nodes cannot represent one individual. Labels *v*∈*V* are classified as:

- *I*_1_, *I*_2_,…, *I_r_* are *identifier* attributes such as *name* and *SSN*;
- *Q*_1_, *Q*_2_,…, *Q_q_* are *quasi-identifier* attributes such as *zip* and *sex* that may be obtained by an adversary from external sources and used for re-identification of individuals;
- *S* is a *sensitive* attribute such as *diagnosis* or *income* that is assumed to be unknown to adversaries.

Identifier attributes are removed from published social-network data, but quasi-identifier and sensitive attributes as well as the graph structure are usually released. Sensitive attributes as well as structural properties such as in-degree, out-degree, and existence of a relationship between two entities represent private information that should be protected. Unfortunately, there are multiple techniques that an adversary can use to disclose private information. As pointed out in the microdata privacy literature, an adversary may use record-linkage techniques between quasi-identifier attributes and external available information to glean the identity of individuals [41]. We consider in-degree, out-degree and the sensitive attribute of nodes to be of the same nature as other traditional quasi-identifier attributes. So, an adversary may have knowledge about some characteristics of the victim, such as quasi-identifier attributes, in-degree, out-degree and the sensitive attribute.

Some structural properties such as degree and sensitive value belong to both background knowledge of adversary and private information. In contrast to reality, most existing works assume a separation between private information and background knowledge but this assumption is not applied in this paper. To overcome this problem, we limit the certainty of new extra knowledge about the sensitive attribute, degree, and relationships, in case the adversary has some knowledge about the target.

Now, we define privacy constraints for the above-mentioned private information in the data model of Definition.1 (considering (*α*, *β*)-privacy [17] and *l*-diversity [11] privacy models for relational data). In privacy-preserving social-network publications, the data publisher publishes the anonymized version of *N*, denoted by N*. The published network (N*) should protect the private information of all individuals of the original network data *N*. Besides the above private information, we also protect against membership disclosure with respect to known quasi-identifiers, which reduces the certainty of finding extra information from the published network.

To address presence privacy, we define α-presence as in [17]. We use Pr(*i*∈*N*) to denote an adversary’s knowledge about the probability of existence of an individual *i* (with specified quasi-identifier attributes) in the original network data.

**Definition 2:** (*α-Presence*): Given a network data *N*, let N* be its anonymized version. N* satisfies α-presence, if for each individual *i* with specified quasi-identifier values (*i*[*QIds*] = {*q*_1_, *q*_2_,…, *q_q_*}) that is possible *i*∈N* then Pr(*i*∈*N*)≤*α*.

For sensitive-attribute-association privacy, we define *β*-sensitive association as the adversary's belief of an association between individuals and value of sensitive attribute S as in [17] [11]. We use (*i*, *s*) to denote an association between an individual *i* with certain specific attributes (some values of an individual’s characteristics are known) and a sensitive value *s*. Since the inference of any private association of a specific individual is based on presence of his/her node in the original network, we define the sensitive-association privacy probability as conditionally dependent on the presence privacy probability. Specifically, we use Pr((*i*, *s*)∈*N*|*i*∈*N*) to denote the adversary's belief in probability of association (*i*, *s*) in *N*, with the assumption that the node of individual *i* exists in *N*.

**Definition 3:** (*β-sensitive association*): Given a network data *N*, let N* be its anonymized version. N* satisfies *β*-sensitive association, if for each individual *i* with some specific attributes (quasi-identifiers, in-degree, out-degree) that there is an association to sensitive value *s* in N* ((*i*, *s*)∈N*), the conditional probability of the existence of that association in *N* should be under *β*(Pr((*i*, *s*)∈*N*|*i*∈*N*)≤*β*).

We add degree-association and relationship constraints to (*α*, *β*)-privacy [17] to extend it for network data as follows.

To protect degree-association, we define *γ*-degree association as an adversary's belief of the association between an individual *i* with some specific attributes and his/her in-degree (or out-degree) in the network data. We use (*i*, *d*) to denote the association between an individual *i* and an in-degree (or out-degree) *d*. As with sensitive-association, we define degree-association privacy probability as conditionally dependent on presence privacy probability.

**Definition 4:** (*γ-degree association*): Given a network data *N*, let N* be its anonymized version. N* satisfies *γ*-degree association if for each individual *i* with some specific attributes (quasi-identifiers, sensitive) that there is an association to in-degree (out-degree) value *d* in N* ((*i*, *d*)∈N*), the conditionally probability of existence of that association in *N* should be under *γ γ*(Pr((*i*, *d*)∈*N*|*i*∈*N*)≤*γ*).

Finally, for relationship privacy, we define δ-relationship as an adversary's belief of existence of a directed edge from one individual to another in the network. We use (*i*_1_, *i*_2_) to denote the directed relation from individual *i*_1_ *to i*_2_. Similarly, we define relationship privacy probability as conditionally dependent on presence privacy probability of both individuals.

**Definition 5:** (*δ-relationship*): Given a network data *N*, let N* be its anonymized version. N* satisfies *δ*-relationship if for each of two individuals *i*_1_ and *i*_2_ with some specified characteristics, there is a directed relationship between them in N* ((*i*_1_, *i*_2_)∈N*), then Pr((*i*_1_, *i*_2_)∈*N*|*i*_1_∈*N*, *i*_2_∈*N*)≤δ.

Based on the above definitions, we now define (*α*, *β*, *γ*, *δ*)-*SNP* (Social Network Privacy):

**Definition 6:** ((*α*, *β*, *γ*, *δ*)-*SNP*): Given a network data *N*, let N* be its anonymized version. N* satisfies (α,β,γ, *δ)-SNP* if it satisfies all *α*-presence, *β*-sensitive-association, *γ*-degree-association, and *δ*-relationship requirements.

Now, we express the necessity of defining (*α*, *β*, *γ*, *δ*)-*SNP*. In all network data anonymization based on *k-*anonymity [6,9,4,5,8], the number of candidate nodes for each query based on background knowledge is greater than *k*. In this situation, an adversary cannot detect which of these candidate nodes is the victim. However, if all of these nodes have the same properties then an adversary can identify those properties of a victim. All methods that satisfy only *k-*anonymity requirement only protect against re-identification attacks, but they do not protect against private information disclosure. (*α*, *β*, *γ*, *δ*)-*SNP* limits disclosure of each piece of private information under specified thresholds *α*, *β*, *γ*, and *δ*.

*l-*diversity [11] was proposed for tabular data to address drawbacks of k-anonymity. In distinct *l-*diversity, sensitive values of each group must at least have at least *l* distinct values. Distinct *l-*diversity does not protect probabilistic inference attacks, because frequency of each sensitive value is not controlled. Entropy *l-*diversity was proposed to remove this problem [11]. (*α*, *β*, *γ*, *δ*)-*SNP* does not limit the number of distinct values of private information but limits probability of assigning the most frequent value to nodes of each group.

The *k-*anonymity and *l-*diversity models only preserve one private attribute of each node [16]. However, in real-world social-network data, there is lots of private information. (*α*, *β*, *γ*, *δ*)-*SNP* extends *l-*diversity for three kinds of private information (sensitive attribute, degree, and relationships) with different thresholds. In addition, it protects against membership disclosure. Different thresholds allow us to determine the importance of disclosure of each type of private information, dependent on application.

## ASN

In this section, we present the anonymization technique *ASN* for (*α*, *β*, *γ*, *δ*)-SNP. Initially, all individuals are partitioned into separate groups (as explained in the following section). Removing identifiers prior to publication fails to fully protect privacy. As stated, most anonymization algorithms use generalization-based techniques that often result in a considerable amount of information loss. In this paper, instead of generalizing values and nodes, exact quasi-identifier values, sensitive values, nodes degree, and successor nodes of individuals of each group are published in separate *tables*. The only common (repeated) attribute in all tables is group ID. By lossy join of these tables based on their group ID, every individual will be associated with all distinct sensitive values, all degree values, and all successor nodes of his or her group (i.e., values are permutated). This issue injects uncertainty in association of sensitive, degree and relationship to each individual.

Compared to the generalization-based technique, publishing the exact quasi-identifier values increase the accuracy of the results of aggregate queries. For example, one method for generalizing numerical values (such as age) is to use range values. In this situation, if we want to count all individuals in network data who are 20 years old, we should sum the probability of 20 in all the range-values which contain 20. Uniform distribution is assumed for this computation; in reality this is inaccurate. In addition, some generalized values may not exist in the original data. For example, if the age range of a group with four members is [20, 60], it covers 40 distinct values with a probability of $\frac{1}{10}$, while in the original data there are only four different values. This assumption decreases data utility for generalization-based techniques.

However, publishing the exact quasi-identifier values does not protect against presence disclosure. In order to protect presence privacy, associations between quasi-identifier attributes values of each individual are broken by publishing them in different tables. The number of individuals generated by lossy join of these tables is more than the number of individuals in the original data. In other words, some false individuals are generated by lossy join, which creates uncertainty about the membership of each person in the original data.

We formulate *ASN* below:

**Definition 7:** (*ASN*): Given a social network data *N* = (*V*, *E*), assume that all identifier attributes *I*_1_, *I*_2_,…, *I_r_* are removed and one unique and random label *l* is assigned to each vertex *v*. Vertices are partitioned into *n*′ groups *G* = {*G*_1_, *G*_2_,…, *G*_n′_} such that $\overset{n'}{\underset{i\;=\;1}{⋃}}G_{i}\;=\;V$, and for any *i*≠*j*, *G_i_*∩*G_j_* = ∅. ASN produces k quasi-identifier auxiliary tables (QATs), a sensitive table (ST), a degree table (DT), and a successor vertices table (SVT) so that:

- Quasi-identifier attribute set *Q* = {*Q*_1_.*Q*_2_,…, *Q_q_*} is partitioned into *k*(2≤*k*≤*q*) sets *P* = {*P*_1_,…, *P_k_*} such that $\forall i,j:\;P_{i}\cap P_{j}\;=\;\emptyset \;and\;\overset{k}{\underset{i\;=\;1}{⋃}}P_{i}\;=\;Q$. Each set *P_i_* = {*Q_i_*,_1_, *Q_i_*,_2_,…, *Q_i|Pi|_*} (*|P_i_|* denotes the size of *P_i_*) corresponds to an auxiliary table QAT_i_ of schema (*GID*, *Q*_i,1_,…, *Q_i_*,_|Pi|_,count) with *|P_i_|+*2 columns. Furthermore, for any group *G_j_*(1≤*j*≤*n′*) and any distinct quasi-identifier values (*q*_1_, *q*_2_,…, *q_|Pi|_*) of (*Q_i_*,_1_, *Q_i_*,_2_,…, *Q_i|Pi|_*) in $G_{j}$, there is a tuple (*j*, *q*_1_, *q*_2_,…, *q_|Pi|_,.c*)∈*QAT_i_*, where *j* is the ID of the group, and *c* is the number of vertices *v*∈*G*_j,_ such that $v[Q_{i,1}]\;=\;q_{1},\;v[Q_{i,2}]\;=\;q_{2},\;\ldots ,\;v[Q_{i,|P_{i}|}]\;=\;q_{\left|P_{i}\right|}\;(v[A]$ denotes the value of properties or attributes *A* of vertex *v*).
- The sensitive attribute S corresponds to a sensitive table *ST*(*GID*, *S*, *count*). Furthermore, for any group *G_j_*(1≤*j*≤*n′*) and any distinct sensitive value *s* of *S* in *G*_jj_, there is a tuple (*j*, *s*, *c*)∈*ST*, where *j* is the ID of the group, and *c* is the number of vertices *v*∈*G_j_* such that *v*[*S*] = *s*.
- The exact in-degree and out-degree of vertices are published to a degree table *DT*(*GID*, *label*, *Din*, *Dout*). Furthermore, for any group *G_j_*(1≤*j*≤*n′*) and any vertex *v* in *G_j_*, there is a tuple (*j*, *l*, *din*, *dout*)∈*DT*, where *j* is the ID of the group, and *din*, *dout* are the in-degree and out-degree v such that *v*[*label*] = *l*.
- Successor vertices of nodes of each group are published to a successor vertices table *SVT*(*GID*, *label*, *Dout*). Furthermore, for any group *G_j_*(1≤*j*≤*n′*) and any distinct vertex *u* in successor nodes of *G_j_*, there is a tuple (*j*, *l*, *c*)∈*SVT*, where *j* is the ID of the group, and *c* is the number of vertices *v*∈*G_j_* such that *u*∈*v*[*Successor*] and *u*[*label*] = *l*.

As stated above, to protect presence privacy, we publish quasi-identifiers in *k* QAT tables, where 2≤*k*≤*q* (*q* is the number of attributes of quasi-identifiers). Greater *k* causes more presence privacy and less information loss, because lossy join of more QATs generates more extra records. We show in the following sections that the time complexity to measure presence privacy grows exponentially as *k* increases. So, to protect presence privacy, it is sufficient to set *k* to 2. However, utility of data is better preserved since the number of false individuals generated by lossy join decreases when *k* equals 2.

### Example

Here an example of the ASN scheme is presented. Fig 1 illustrates the original network of money transformation. Each node contains five labels, categorized as: *name* (identifier), {*sex*, *job*, *zipcode*} (quasi identifier), and *income* (sensitive).

**Fig 2:**
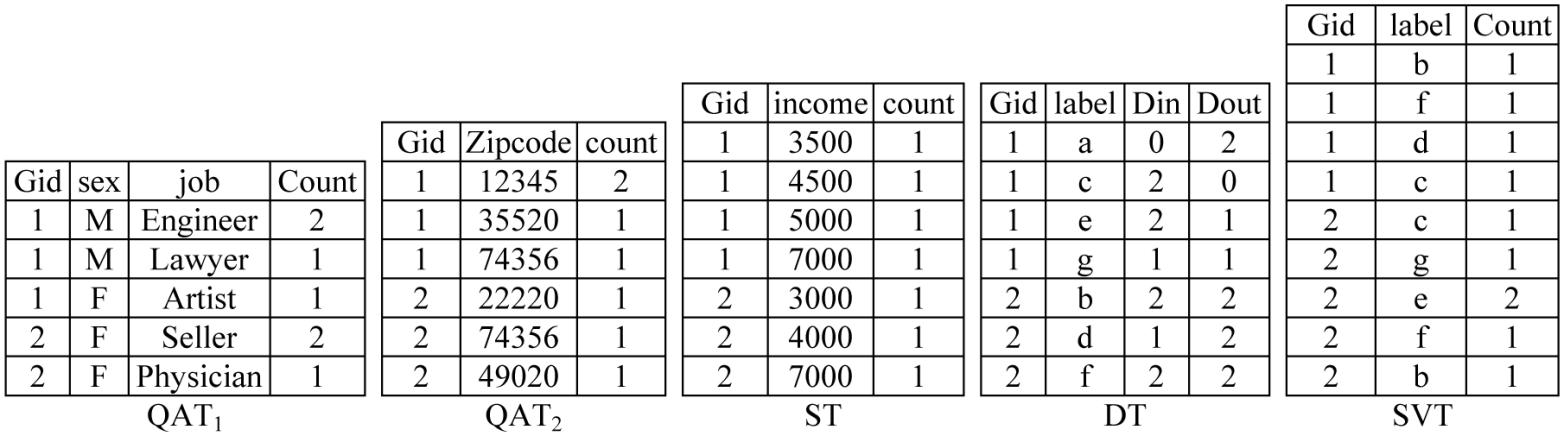
An example of *ASN*.

Fig 2 illustrates an example of an ASN on the network data in Fig 1. The name attribute of each node is removed and replaced with a random label (first letter of its name). We split nodes into two groups *G*_1_(*Amin*, *Cirus*, *Emad*, *Goli*) and *G*_2_(*Bahar*, *Diba*, *Farah*).

In this example, *k* is equal to 2. Quasi-identifier attributes are partitioned into two sets {*sex*, *job*} and {*zipcode*} (we provide a greedy algorithm for partitioning the quasi-identifier attributes set in the following section). So, we publish the anonymized network into five tables. *QAT*_1_, *QAT*_2_, ST, DT, and SVT, where group ID (Gid) is the common attribute in all of them.

*ASN* creates a table *QAT*_1_ that contains exact values of attributes (*sex*, *job*) with their frequency counts in each group. For example, these values for both *Amin* and *Cirus* in *G*_1_ are (*M*,Engineer). So its frequency count in *G*_1_ is equal to 2. Similarly, *QAT*_2_ and ST contain values of *zip code* and sensitive value, respectively, with their frequency counts in each group. DT contains random labels and exact in-degree and out-degree values of nodes in each group. For example the first tuple of DT (1, *a*,0,2) in Fig 2 means that there is one node with label *a* in *G*_1_ with in-degree and out-degree 0 and 2, respectively. SVT contains labels of successor nodes and frequency counts for each group. The tuple (2, *e*,2) means that node *e* is the successor of two nodes in *G*_2_ (*Emad* is the successor *Bahar*, *Diba* in Fig 1).

## Measurement of Privacy Constraints in ASN

Partitioning individuals into groups by anonymization algorithm has a significant impact on the level of privacy requirements for published data in ASN. In this section, we show how to measure privacy parameters of individuals for each group, as well as the overall released network data. The anonymization algorithm uses these measuring methods to generate groups that satisfy the privacy requirements of (*α*, *β*, *γ*, *δ*)-*SNP*.

**Lemma 1:** *(covering groups)*: A victim *i* belongs only to one of the multiple groups G* = {*G*_1_,…, *G_k_*} that cover *i* based on background knowledge of the adversary.

**Proof**: There are multiple groups G* that cover *i* with specified background knowledge about an individual. By Definition 1, there are no multiple nodes related to one individual in the network data. Furthermore, by Definition 7, the intersection between groups in ASN is empty. Therefore, individual *i* belongs only to one G_j_.

For example, if an adversary knows that *sex* and *zip code* of *Diba* are *F* and 74356, respectively, based on the published data in Fig 2, he may belong to *G*_1_ and *G*_2_. But, in reality he can only be in one of those groups. As a result, if there are multiple groups that cover *i*, and we want to measure the probability of some disclosure for *i*, it will be less than the maximum probability for those groups.

### Measurement of Presence Privacy

**Fig 3:**
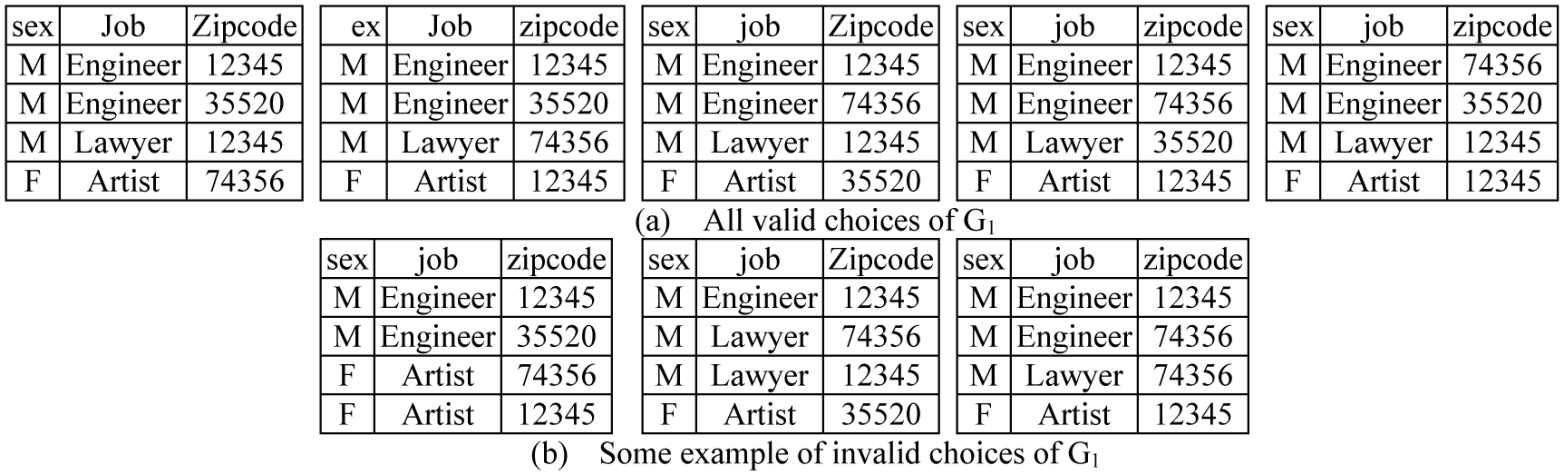
All valid choices and some invalid choices for reconstructing group *G*_1_ from Fig 2.

*ASN* hides presence of individuals by breaking associations between quasi-identifier values. When an adversary tries to reconstruct quasi-identifier values, he/she will have multiple candidates due to lossy join of QATs. For example, by lossy join of QATs on Gid in Fig 2, an adversary can generate 3 × 3 = 9 distinct quasi-identifier values (*possible combinations*) (because *G*_1_ corresponds to 3 tuples in *QAT*_1_ and 3 tuples in *QAT*_3_), some of which are false and do not exist in original network data. For example, the tuple (*M*, *Engineer*,74356) is a false one based on Figs 1 and 2. Regarding count attributes, he/she knows that there are 4 nodes in *G*_1_. In addition, there are no two nodes with equal quasi-identifier values. There are 94 choices, but since frequency of each value is specified in QAT, some of them are not valid choices and do not match with their frequency (*count* column) in QATs. Fig 3(a) illustrates all *valid choices* (VC) for group 1 (5 choices), and Fig 3(b) shows some invalid choices (in the left table (*F*, *Artist*) appears in two tuples while its *count* value in *QAT*_1_ is 1). Quasi-identifier values related to *Amin* (*M*, *Engineer*,12345) and *Cirus* (*M*, *Engineer*,35520) appear in 4 and 3 cases of valid choices, so their presence probabilities are $\frac{4}{5}$ and $\frac{3}{5}$, respectively.

To measure presence probability, we count valid choices that contain probable combination (*selective valid choices* (SVC)) and divide it by all valid choices. In the above example the max presence probabilities are $\frac{4}{5}$ and $\frac{2}{3}$ in *G*_1_ and *G*_2_, respectively. Based on Definition 2, to achieve α-presence, we should restrict max presence probability of each group below α. Therefore, the published network data in Fig 2 satisfied $\frac{4}{5}$-presence.

**Theorem 1:** (*Presence Privacy*): Let N be a network data and N* be its anonymized network by ASN, with tables N* = {*QAT*_1_, *QAT*_2_, *ST*, *DT*, *SVT*}. Let G* = {*G*_1_,…, *G_k_*} be the set of groups that cover an individual *i* based on quasi-identifier values. Maximum presence privacy for *i* is $\text{Pr}\left(i\in N\right)\leq {\text{max}}_{1\leq j\leq k}\left\{\frac{\left|SVC(G_{j})\right|}{\left|VC(G_{j})\right|}\right\}$.

**Proof:** Based on Lemma1 individual *i* belongs to only one group of G*, so Pr(*i*∈*N*)≤*max*_1≤j≤k_{Pr(*i*∈*G_j_*)}. Based on *count* columns in QATs, adversaries have multiple choices to reconstruct group members of *G*_jj_ (*VC*(*G_j_*)). Some of these valid choices contain *i*’s quasi-identifiers. So, Pr(*i*∈*G_j_*) is equal to the number of VC containing *i*, divided by the total number of VC. The max probability is related to a combination of the most frequent value of each QAT in *G_j_* (*probable combination* (*PC*)). *Selective valid choice*s set *G_j_*(*SVC*(*G_j_*)) shows valid choices that contain PC. Thus, $\text{Pr}\left(i\in G_{j}\right)\leq \frac{\left|SVC(G_{j})\right|}{\left|VC(G_{j})\right|}$. Therefore $\text{Pr}\left(i\in N\right)\leq {\text{max}}_{1\leq j\leq k}\left\{\frac{\left|SVC(G_{j})\right|}{\left|VC(G_{j})\right|}\right\}\;$

Since frequency of each distinct value in each QAT is known, and selection of each combination depends on previously selected combinations, we cannot find a specific equation to compute the number of valid choices. Instead, we develop a recursive function that uses greedy and dynamic programming techniques to count all *valid choices* of each group in the following section.

With the same group size, the number of possible combinations increases *exponentially* w.r.t number of QATs. For example, if in Fig 2 we split quasi-identifier attributes in 3 QATs, and we have 2×3×3 = 18 combinations in *G_j_* from lossy join of QATs. As a result, the number of valid choices also increases exponentially. In this paper, we consider only two QATs to decrease execution time and information loss. However, increasing possible combinations serves to decrease presence probability. So, we develop an algorithm to partition quasi-identifier attributes so that the number of possible combinations is maximal.

#### Presence probability algorithm

**Fig 4:**
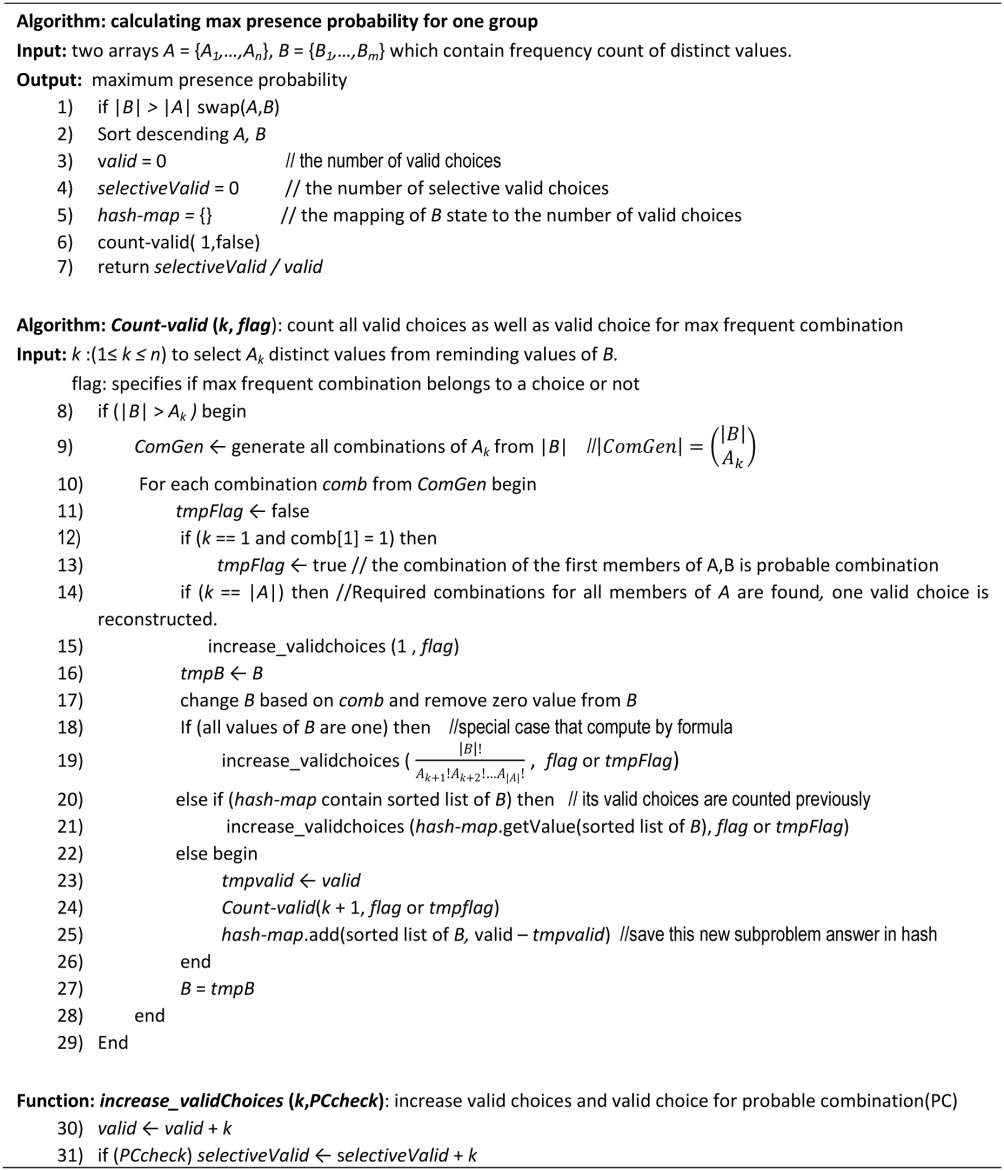
Recursive function for computing presence probability.

Here we present a recursive function to count all valid and selective valid choices for computing presence probability of one group (Fig 4).

Inputs of this algorithm are two arrays: *A*, *B*. These arrays are the count column of two QATs for each group. *A_i_* denotes frequency of distinct value *i* (similar for *B*). Their summation shows group size: ($\sum_{i\;=\;1}^{\left|A\right|}A_{i}\;=\;\left|G\right|\;=\;\sum_{i\;=\;1}^{\left|B\right|}B_{i}\;=\;\left|G\right|$). Values of arrays *A* and *B* are [2,1] and [1,1,1] for *G*_2_ in Fig 2, respectively.

The general trend of the algorithm *count-valid is*: in each step *k*, choose *A_k_* distinct values from *B*. There are BAk possible choices (|*B*| is the number of distinct values in *QAT*_2_ or size of *B*) to combine with *k*th attribute value in *A* with frequency *A_k_* (line 9). For each possible choice (lines 10–28), the decrement value of *B_j_* is based on selected values, and if some values of *B* are zero, remove them from *B* (line 17). Continue with *k*+1 in *A* (line 24) until *k* = |*A*| (line 14). If there is no possible choice (*A_k_*>|*B*|), do not continue for *k*+1 and return to *k*-1 to check other choices (line 8).

Execution time is less than GA1,A2,…,A|A|=GA1G-A1A2…G-A1-…-A|A|-1A|A|=G!A1!A2!…A|A|!, since |*B*| may be less than |*G*|.

The following techniques are applied to improve execution time:

- We choose B as the smallest set to decrease branch factor of the search-space tree (line 1). In each step *k*, we call the recursive function BAk times (lines 9–10) for the next step *k*+1.
- We sort *A* in descending order (line 2), since in each step we decrement *A_k_* distinct values of *B*. After that, the number of reminding distinct values of *B* reduces. So, if we first choose higher values of *A_k_*, |*B*| reduces rapidly. Thus pruning of the search tree is done in its higher levels.
- In the special cases where |*B*| = |*G*| or all values of *B_j_* are equal to 1, the number of valid choices equals $\frac{\left|B\right|!}{A_{1}!A_{2}!\ldots A_{\left|A\right|}!}$ (There are |*B*| choices for *A*_1_, |*B*|-*A*_1_ choices for *A*_2_…). Since array *B* changes in each step, when we reach the situation where all remaining *B_j_* = 1 in step *k*, we do not continue, and add $\frac{\left|new\;B\right|!}{A_{k}!A_{k+1}!\ldots A_{\left|A\right|}!}$ to valid choices (lines 18–19).
- Last but not least, we use a dynamic programming technique to significantly reduce execution time. Values of *A* remain unchanged in this recursive algorithm, but *B* changes in each step and $\sum_{j\;=\;1}^{\left|B\right|}B_{j}\;=\;\sum_{i\;=\;k}^{\left|A\right|}A_{i}$. So, when we compute the number of valid choices for one *B*, its result is the same for all permutations of elements of *B* with respect to the same *A*. Since array *A* remains unchanged in the recursive function, the state of *B* determines the next subproblem. We store the state of *B* and the number of its valid choices in one hash map (line 25) (complexity of insert and search is O(1)). When we reach this step again, we do not repeat the process of counting valid choices, but use the saved cases (lines 20–21). The number of repetitive cases is very high so size of the hash map does not grow rapidly and runtime is dramatically decreased.

#### Partitioning algorithm for quasi-identifier attributes

Here, we present a simple greedy algorithm to partition quasi-identifier attributes into two sets, *S_1_* and *S_2_*, and store distinct values of attributes of each set *S_i_* in table *AT_i_*. The purpose of this algorithm is to maximize the number of tuples generated by lossy join of *AT_i_*s. The number of these tuples is equal to the product of distinct values of each set. For *q* quasi-identifier attributes, there are 2*^q^*-2 states to split them into two sets. But, we use a greedy algorithm to find a partitioning with the maximal product of numbers of distinct values of all partitions.

Let *d_s_* be the number of distinct values of attributes of set *S* in network data. For example *d*_{sex,job}_ in Fig 1 is 5, because we have 5 distinct values for (*sex*, *job*) in the table. First, we sort attributes {*Q*_1_,…, *Q_q_*} based on *d*_{i}_; this means that *Q*_1_ has minimum and *Q_m_* has maximum distinct values in the network data. Then, we put *Q*_1_ in *S*_1_ and *Q*_2_ in *S*_2_. Now we add *Q_i_(3*≤*i*≤*q)* as follows: compare $d_{S_{1}\cup \{Q_{i}\}}\times d_{S_{2}}$ and $d_{S_{1}}\times d_{S_{2}\cup \{Q_{i}\}}$ and add to the part with maximum product.

### Measurement of Sensitive-Attribute-Association Privacy

We now describe how to measure sensitive-attribute-association privacy, stated in Definition 3 as a conditional probability Pr((*i*, *s*)∈*N*|*i*∈*N*). It is the probability that individual *i* with specific quasi-identifier values and degree in *N*, associates to sensitive value s. For example *Amin* with quasi-identifier (F, Engineer, 12345) and in-degree 0 may exist in $G_{1}.\;P\left(\left(Amin,\;3500\right)\in N|Amin\in N\right)\;=\;\frac{1}{4}\;$ because frequency count of 3500 in *G*_1_ is 1 and group size is 4 (Fig 2). As association is broken between the sensitive attribute and others by publishing data in different tables, adversary’s background knowledge about individual *i* shows him/her in multiple groups (knowledge about a victim matches with possible combinations in more than one group), while in reality he/she belongs to only one group. So the probability of associating sensitive value *s* to that victim is below the maximum probability of sensitive association between all groups that *i* may belong to.

**Theorem 2:** (*Sensitive-Association Privacy*): Let N be a network data and N* be its ASN-anonymized network with tables N* = {*QAT*_1_, *QAT*_2_, *ST*, *DT*, *SVT*}. Let G* = {*G*_1_,…, *G_k_*} be the set of all groups that cover *i* based on quasi-identifiers and degree values. Maximum sensitive-association privacy is $\text{Pr}\left(\left(i,s\right)\in N|i\in N\right)\leq {max}_{1\leq j\leq k}\{\frac{c_{j}}{\left|G_{j}\right|}\}$ where *c_j_* is the maximum frequency count of sensitive values in group *G_j_*.

**Proof:** Based on Lemma1 individual *i* belongs to only one group of G*, So Pr((*i*, *s*)∈*N|i*∈*N*)≤*max*_1≤j≤k_{Pr((*i*,s)∈*G_j_*|*i*∈*G_j_*)}. But when *s* is the most frequent sensitive value in group *G_j_*, then the maximum sensitive-association probability occurs for (*i*,s) in that group. If the frequency of the most frequent sensitive value in *G_j_* is *c_j_*, and |*G_j_*| is the size of group *G_j_*, then $\text{Pr}\left(\left(i,s\right)\in G_{j}|i\in G_{j}\right)\leq \frac{C_{j}}{G_{j}}$. Thus $\text{Pr}\left(\left(i,s\right)\in N|i\in N\right)\leq {max}_{1\leq j\leq k}\left\{\frac{c_{j}}{\left|G_{j}\right|}\right\}$.

In Fig 2, maximum sensitive-association probabilities for *G*_1_ and *G*_2_ are $\frac{1}{4}$ and $\frac{1}{3}$, respectively. To achieve *β*-sensitive-association, we should restrict maximum sensitive-association probability of each group to below *β*. The published network in Fig 2 satisfies $\frac{1}{3}$-sensitive-association privacy.

### Measurement of Degree-Association Privacy

The method of measuring for degree-association privacy is similar to that used for sensitive-attribute association. As shown in Definition 4, it is a conditional probability Pr((*i*, *d*)∈*N|i*∈*N*). This describes the probability that in-degree (out-degree) of individual *i* is *d*, if the adversary knows individual *i* exists in *N* with specific background knowledge (quasi-identifier or sensitive values). For example, the adversary knows that data of *Amin* with quasi-identifiers (*M*, *Engineer*,12345) exists in the published data; based on his quasi-identifier values, she should only be in $G_{1}.\;P\left(\left(Amin,0\right)\in N|Amin\in N\right)\;=\;\frac{1}{4}$ because number of appearances of 0 in the *Din* column of DT for group G_1_ is 1, and group size of G_1_ is 4 (Fig 2).

**Theorem 3:** (*Degree-Association Privacy*): Let *N* be a network data and N* be its ASN-anonymized network with tables N* = {*QAT*_1_, *QAT*_2_, *ST*, *DT*, *SVT*}. Let G* = {*G*_1_,…, *G_k_*} be the set of all groups that cover *i* based on quasi-identifiers and sensitive values. The maximum probability of assigning degree *d* as in-degree (out-degree) is $\text{Pr}\left(\left(i,d\right)\in N|i\in N\right)\leq {max}_{1\leq j\leq k}\{\frac{{cin}_{j}}{\left|G_{j}\right|}\}$ ($\text{Pr}\left(\left(i,d\right)\in N|i\in N\right)\leq {max}_{1\leq j\leq k}\{\frac{{cout}_{j}}{\left|G_{j}\right|}\}$), where *cin_j_* is the frequency of the most frequent in-degree in group *G_j_*, (*cout_j_* is the frequency of the most frequent out-degree in *G_j_*).

**Proof:** Based on Lemma 1 individual *i* belongs to only one group of G*, so Pr((*i*, *d*)∈*N|i*∈*N*)≤*max*_1≤j≤k_{Pr((*i*,d)∈*G_j_*|*i*∈*G_j_*)}. For each group *G_j_*, the probability of assigning in-degree *d* to *i* equals the number of appearances of *d* in *Din* columns of DT divided by the group size of *G_j_*. The maximum probability in *G_j_* occurs when *d* is the most frequent in-degree in that group. So $\text{Pr}\left(\left(i,d\right)\in G_{j}|i\in G_{j}\right)\leq \frac{{cin}_{j}}{\left|G_{j}\right|}$. As a result $\text{Pr}\left(\left(i,d\right)\in N|i\in N\right)\leq {max}_{1\leq j\leq k}\{\frac{{cin}_{j}}{\left|G_{j}\right|}\}$.

The proof is similar for out-degree.

In Fig 2, maximum in-degree-association probabilities for *G*_1_ and *G*_2_ are $\frac{2}{4}$ and $\frac{2}{3}$, respectively. So, the published network data satisfies $\frac{2}{3}$-in-degree-association privacy. In addition, the maximum out-degree-association probabilities for *G*_1_ and *G*_2_ are $\frac{2}{4}$ and $\frac{3}{3}$, respectively. Based on Definition 4, to achieve γ-degree-association, we should restrict the maximum degree-association probability of each group below γ. The published network in Fig 2 satisfies 1-degree-association.

### Measurement of Relationship Privacy

The measuring method for relationship privacy is similar to that used for presence privacy. As shown in Definition 5, it is a conditional probability Pr((*i*_1_, *i*_2_)∈*N*|*i*_1_∈*N*, *i*_2_∈*N*). This describes the probability of a directed relationship from *i*_1_ to *i*_2_, where an adversary knows that individuals *i*_1_ and *i*_2_ exist in *N* with specified background knowledge about quasi-identifiers, sensitive-attribute or degree values. There are some candidate node labels for each *i*_1_ and *i*_2_. In the worst case, there is only one label that covers each victim.

As an example, let an adversary know that *Amin* and *Diba* exist in a data network, and *Amin* has quasi-identifiers (*M*, *Engineer*,12345), in-degree 0 and out-degree 2. Based on quasi-identifier values, he should be only in *G*_1_. Based on his degrees, his only candidate label is *a*. In addition, the adversary knows that *Diba*’s quasi-identifier values are (*F*, *Seller*,74356) and her in-degree and out-degree values are 1 and 2, respectively. Regarding his quasi-identifier values, she should be in G_2_, and based on her degree she matches to label *d* (Fig 2). Since *a* belongs to *G*_1_ and *d* appears in successors of vertices of *G*_1_ (table SVT in Fig 2), it is probable that a directed edge from *a* to *d* exists. The lossy join of DT with SVT shows that there are 4×4 = 16 candidate directed edges {(*a*, *b*), (*a*, *f*), (*a*, *d*), (*a*, *c*), (*c*, *b*), (*c*, *f*), (*c*, *d*), (*c*, *c*), (*e*, *b*), (*e*, *f*), (*e*, *d*), (*e*, *c*), (*g*, *b*), (*g*, *f*), (*g*, *d*), (*g*, *c*)} (there are 4 distinct labels for *G*_1_ in DT and 4 in SVT). By considering the *Dout* column in DT and the *count* column in SVT for *G*_1_, it can be determined that there are 4 directed edges. So there are 164 choices to select 4 out of 16 edges. The *Dout* value of each label in DT specifies how many times that label appears as the source of edges, and the *count* value of SVT specifies how many times that label appears as the destination of edges. Similarly to presence-privacy measuring, some of these choices are invalid. For example {(*a*, *b*), (*a*, *f*), (*c*, *d*), (*e*, *b*)} is not a valid set of edges since the *Dout* of *c* is zero; so it should not be the source of any edges and *b* appears as destination of two nodes; based on SVT it should be the destination of one member of *G*_1_. For this case there are 12 *valid edges choices* (VEC) ({(*a*, *b*), (*a*, *f*), (*e*, *c*), (*g*, *d*)}∈*VEC*(*G*_1_)). Directed edge (*e*, *d*) appears in 3 choices, so the probability of its existence equals $\frac{3}{12}$. (*a*, *b*) is the probable combination edge and its existence probability is $\frac{6}{12}$.

Our goal is to prevent disclosure of edge between two individuals, while the detection of the lack of edge between individuals is easy. In the above example, if node *d* does not exist in possible successors of *a*, we can easily detect that there are no directed edges from *a* to *d*.

Similarly to measuring presence probability, there are two arrays that specify the frequency of each item (the *Dout* column in DT and the *count* column in SVT). We want to measure the maximum probability of disclosure of one edge (combination). In this case the algorithm shown in Fig 4 is used.

**Theorem 4:** (*Relationship Privacy*). Let N be a network data and N* be its ASN-anonymized version with scheme tablesN* = {*QAT*_1_, *QAT*_2_, *ST*, *DT*, *SVT*}. Let $L_{1}\;=\;\{l_{1},\ldots ,\;l_{k_{1}}\}$ and $L_{2}\;=\;\{l_{1},\ldots ,\;l_{k_{2}}\}$ be sets of all node labels that cover *i*_1_ and *i*_2_ based on background knowledge (quasi-identifiers, sensitive and degree values) and G* = {*G*_1_,…, *G_k_*} be all groups covering *L*_1_ nodes such that (∀l∈*L*_1_ ∃*g*∈G*:*l*∈*g*). Then, the probability of disclosure of directed edge from *i*_1_ to *i*_2_ in network data *N* equals $\text{Pr}\left((i_{1},i_{2})\in N|i_{1}\in N,i_{2}\in N\right)\leq {\text{max}}_{1\leq j\leq k}\left\{\frac{\left|SVEC(G_{j})\right|}{\left|VEC(G_{j})\right|}\right\}$.

**Proof:** As stated in Lemma1 each individual *i* corresponds to only one node label. So in computing the relationship probability, the sum of probabilities of multiple cases is not considered as total probability, but their maximum is assumed as the upper bound. Thus $\text{Pr}\left((i_{1},i_{2})\in N|i_{1}\in N,i_{2}\in N\right)\leq {max}_{\forall \;l_{1}\in L_{1},l_{2}\in L_{2}}\left\{\text{Pr}\left((l_{1},l_{2})\in N\right)\right\}$. Since successors of all nodes of each group are clustered to successors of their group, in order to compute Pr((*l*_1_, *l*_2_)∈N) we should compute it based on group *g*, which contains *l*_1_. If the out-degree value of *l*_1_ is zero, or *l*_2_ is not in the list of successors of *g*, this probability is zero. Otherwise, a directed edge may exist from *l*_1_ to *l*_2_. There are some candidate edges from nodes in *g* and successor nodes of *g*. The sum of out-degree values of nodes of group *g* determines the number of edges going out from nodes of *g*. We must select this number of edges from all possible candidate edges, but some of these choices may satisfy the following two constraints: (VEC(*g*): 1): the number of appearances of each node of group *g* as a source equals its out-degree; 2) the number of appearances of each node of successors of *g* as destination equals its count value. So, Pr((*l*_1_, *l*_2_)∈N) equals the number of these valid choices that contain edges (*l*_1_, *l*_2_), divided by the number of valid edges of *g*. The maximum of these probabilities occurs when *l*_1_ has the highest out-degree of *g* and *l*_2_ is the successor with maximum count. The number of valid choices of *g* containing this case is called *selective valid edges choices of g (SVEC(g))*. So, $\text{Pr}\left((l_{1},l_{2})\in N\right)\leq \frac{\left|SVEC(g)\right|}{\left|VEC(g)\right|}$.

As a result, based on all candidate nodes for *i*_1_ and *i*_2$,\;\text{Pr}\left((i_{1},i_{2})\in N|i_{1}\in N,i_{2}\in N\right)\leq {max}_{\forall l_{1}\in L_{1},\;\exists g\in G^{*}:\;l_{1}\in g\;}\frac{\left|SVEC(g)\right|}{\left|VEC(g)\right|}\;=\;{\text{max}}_{G_{j}\in G^{*}}\left\{\frac{\left|SVEC(G_{j})\right|}{\left|VEC(G_{j})\right|}\right\}$_

In Fig 2, maximum relationship probabilities for *G*_1_ and *G*_2_ are $\frac{4}{5}$ and $\frac{24}{36}$, respectively. Based on Definition 5, to achieve δ-relationship, we should restrict the maximum relationship probability of all pair nodes below *δ*. The published network in Fig 2 satisfies the $\frac{4}{5\;}$-relationship.

To reconstruct graph structure from the published data, there are |*VEC*(*G*_j_| choices to reconstruct output edges of group *j*. So, $\prod_{j\;=\;1}^{n'}\left|VEC(G_{j})\right|$ graphs could be reconstructed from published data. This is a strength of this method that there is low probability of reconstructing the original graph by an adversary.

## Privacy Analysis

In this section, we analyse the privacy of the published data based on ASN that all of its groups satisfy privacy requirements of (*α*, *β*, *γ*, *δ*)-*SNP* based on measuring methods of previous section. In our privacy model and ASN there is no definite boundary between private information and an adversary’s background knowledge. In other words, an adversary may have knowledge about some or all quasi-identifier attributes, in-degree, out-degree and sensitive attributes of a victim. Combinations of background knowledge are as follows:

1. *Value of all quasi-identifier attributes*: Let *p*_i_ be the set of all quasi-identifier attributes in *QAT_i_*., *victim*[*p_i_*] denote the values of *p_i_* attributes of the victim. ${QAT}_{i}\left[G_{j}\right]\;=\;\pi_{p_{i}}(\sigma_{Gid\;=\;j}\left({QAT}_{i}\right))$ shows the distinct values of quasi-identifiers of group *G_j_* in *QAT_i_*. An adversary can guess that the victim may belong to G* = {*G_j_*|∀*i*:*victim*[*p_i_*]∈*QAT_i_*[*G_j_*]}. If G* is empty, then the adversary knows that the victim is not in the released network data. However, if G* is not empty, then the adversary is not sure whether or not the victim belongs to the published data because the victim’s quasi-identifier values may be a false combination generated by lossy join on QATs. In addition, there are no two nodes in the network data for one individual so the victim belongs to only one group in G*. If G* has only one member, the probability of the victim being in the released data (*P_presence_*) is smaller than α (published data satisfies *α*-presence). Moreover, since the published data satisfy (*α*, *β*, *γ*, *δ*)-*SNP* privacy requirements, the probability of disclosure of a sensitive, in-degree and out-degree values, and relationships of a victim are smaller than *P_presence_ β,P_presence_ γ,P_presence_ δ*, respectively. If the adversary already knows that the victim is in published data prior to accessing these data, then *P_presence_* = 1.
2. *Value of some quasi-identifier attributes*: similarly to the previous case, an adversary cannot be sure that the victim is in the published data. In addition, since ASN breaks associations between quasi-identifier attributes, even if an adversary knows that the victim exists in the published data, he/she cannot extract other quasi-identifier values of the victim.
3. *quasi-identifier*, *in(out)-degree and sensitive values*: an adversary can guess that the victim may belong to G* = {*G_j_*|∀*i*:*victim*[*p_i_*]∈*QAT_i_*[*G_j_*] *and victim*[*din*, *dout*]∈*DT*[*G_j_*] and *victim*[*S*]∈*ST*[*G_j_*]}, where *DT*[*G_j_*] = *π_din,dout_*(*σ_gid = j_*DT), *ST*[*G_j_*] = π*_s_*(*σ_gid = j_*ST). If G* is empty, then an adversary is sure that the victim is not in the released network data. However, if G* is not empty, an adversary is not sure whether the victim belongs to the published data. In the worst case scenario, if an adversary has background knowledge determining presence of that victim in the released data, G* has only one member, *G_j_*, and only one node in DT for *G_j_* that matches with the in-degree and out-degree of the victim. In this situation, an adversary can re-identify the victim and find the label related to him/her from the published data. In this case, while the label of the victim is identified, the probability of assigning one specified relationship to the victim (unknown information) is smaller than *δ*. All other cases for background knowledge are special cases of this one In each case, the probability of disclosure of unknown information is below that of specified thresholds, because sensitive-attribute and degree are published in separate tables.

In summary, in the proposed framework we do not allow an adversary to discover new information about victims with likelihood higher than specified thresholds.

In the proposed framework, while we preserve privacy at a specified level, we introduce policies to preserve data utility. First, using an anatomization-based technique for quasi-identifiers, we reduce information loss by publishing exact values of quasi-identifiers. Second, data utility is better preserved by smaller QAT tables, as lossy join of smaller QATs generates less false tuples. Third, degree distribution remains unchanged because we publish exact values for in-degree and out-degree. In all existing works [32,6,9,27,4,13,29,26,31,14] degree of nodes changes through network transformation.

In [6,4], if one edge exists between two members of two super nodes, there is an edge between two super nodes in the anonymized graph. When the graph is reconstructed each member of one super node may be connected to each member of other super node. One super node may have several edges to multiple super nodes. Meanwhile in our method, to reduce information loss only destination-node labels of output edges of each group appear in SVT for that group.

In addition, the algorithm that puts nodes in groups to satisfy privacy constraints has an important effect on data utility, because there is less information loss in a smaller group size.

## Algorithm for Grouping Individuals

In this section, we present the greedy algorithm *GroupingASN* for implementing the ASN. The algorithm partitions individuals of network data *N* into non-overlapping groups so that each group satisfies privacy requirements of (*α*, *β*, *γ*, *δ*)-SNP. To reduce information loss we construct small sized groups. First, we define a desirability metric for locating two individuals within a group to reach privacy requirements as soon as possible. We assign desirability weight between each two individuals based on this metric and then apply a greedy algorithm for grouping them. This algorithm tries to make groups with as few members and as many average edge weights as possible.

First we compute desirability weight for each two individuals of network. According to measuring methods of privacy requirements it is most desirable to group individuals with more difference between their attributes, in-degree, and successor nodes. In this way, each group with smaller group size can reach *α*-presence, *β*-sensitive-association, *γ*-degree-association, and *δ*-relationship. So, we assign higher weights to those pairs that have higher numbers of different properties. Desirability weight of each two individuals is computed as:

$$Dw\left(i,j\right)\;=\;\sum_{k\;=\;1}^{q}\left(F_{QI}\times not\_equal\left(v_{i}\left[{QI}_{k}\right],v_{j}\left[{QI}_{k}\right]\right)\right)+F_{s}\times not\_equal\left(v_{i}\left[S\right],v_{j}\left[S\right]\right)+F_{Din}\times \frac{\left|v_{i}\left[Din\right]-v_{j}\left[Din\right]\right|}{max\left(Din\right)-min(Din)}+F_{Dout}\times \frac{\left|v_{i}\left[Dout\right]-v_{j}\left[Dout\right]\right|}{max\left(Dout\right)-min(Dout)}+F_{succ}\times \left(1-\frac{max\left(v_{i}\left[Dout\right],v_{j}\left[Dout\right]\right)}{\left|v_{i}\left[succ\right]\cup v_{j}\left[succ\right]\right|}\right)$$

In *DW*(*i*, *j*), the function *not-equal* checks equality of arguments: not_equal(x,y)=0x=y1x≠y.

In-degree is numerical and should be protected against an adversary. Grouping individuals with higher differences provides better protection against in-degree disclosure. In our metric, we modify the not-equal function for in-degree to $\frac{\left|v_{i}\left[Din\right]-v_{j}\left[Din\right]\right|}{max\left(Din\right)-min(Din)}$, where max(Din) and min(Din) are maximum and minimum in-degree in network data *N*, respectively. The greater difference in in-degree relative to the range of in-degree in the dataset is more desirable.

Similarly to in-degree, we use $\frac{\left|v_{i}\left[Dout\right]-v_{j}\left[Dout\right]\right|}{max\left(Dout\right)-min(Dout)}$ for out-degree. If two individuals *v_i_* and *v_j_* are located in one group, there are |*v_i_*[*succ*]∪*v_j_*[*succ*]| distinct labels in SVT for that group. The low bound for the probability of the existence of a directed relationship from *v_i_* to each member of |*v_i_*[*succ*]∪*v_j_*[*succ*]| is approximately $\frac{v_{i}\left[Dout\right]}{\left|v_{i}\left[succ\right]\cup v_{j}\left[succ\right]\right|}$. So, the fraction $\frac{max\left(v_{i}\left[Dout\right],v_{j}\left[Dout\right]\right)}{\left|v_{i}\left[succ\right]\cup v_{j}\left[succ\right]\right|}$ approximates the maximum probability of disclosure of directed relationship if these two individuals are put into one group. Lower values express more desirability for placement in one group. So, we use $\left(1-\frac{max\left(v_{i}\left[Dout\right],v_{j}\left[Dout\right]\right)}{\left|v_{i}\left[succ\right]\cup v_{j}\left[succ\right]\right|}\right)$ in the desirability metric. *F_QI_*, *F_S_*, *F_Din_*, *F_Dout_* and *F_SUCC_* are coefficients for quasi-identifiers, sensitive-attribute, in-degree, out-degree, and relationship, respectively. They have important effects on *DW* and grouping methods. Important factors to set these coefficients include thresholds *α*, *β*, *γ* and *δ*. For example, when the constraint *β*-sensitive-association is limited and the density of sensitive-attribute is high and near to *β*, it is better to choose a higher value for *F_S_*. In this way, we increase the effect of such differences in sensitive values of individuals. Our means from density of *x* is the ratio of the number of individuals with the most frequent value for property *x* to the number of individuals in the network data.

First, all individuals locate into ungrouped list. In our grouping method, groups are generated in order. Individuals are added to one group until privacy requirements of its members are satisfied. The first member of each group *k* is the first ungrouped individual. For the next members, in each step an ungrouped individual *t* with maximum desirability weight average with all current members of group *k $(\frac{1}{\left|G_{k}\right|}\sum_{\forall r\in G_{k}}DW[t,r]$*) is selected. When privacy requirements are satisfied for the current group *k*, the next group, *k*+1, is generated. Generation of groups continues until no ungrouped individual remains. If the last group does not satisfy (*α*, *β*, *γ*, *δ*)-*SNP* constraints, it is deleted and its nodes are added to the previously generated groups. For each node in this last group, we find the group with the maximum desirability weight average, so that adding this new node to it does not violate (*α*, *β*, *γ*, *δ*)-*SNP* privacy requirements.

## Simulation Results

In this section, we conduct experiments on two following network datasets to evaluate data utility of anonymized network data generated by our framework:

- URVEmail [42], which contains edges of e-mail exchanges between members of the University of Rovirai Virgili (Tarragona). This email network contains 1133 nodes and 10,933 directed edges (S1 Dataset).
- Random, which is generated by Pajek [43] with 2000 nodes. It contains 109,832 edges (S2 Dataset).

As nodes should contain quasi-identifiers and a sensitive label, we use the micro-dataset Census [44] that, which contains personal information of 1,000,000 American people. Our datasets contain seven quasi-identifiers (age, gender, martial status, race, birth place, education, work class) and one sensitive attribute (salary). We selected 1133 and 2000 tuples randomly to assign to network nodes and set *α* = 0.25, *β* = 0.25, *γ* = 0.7, *δ* = 0.75.

**Fig 5:**
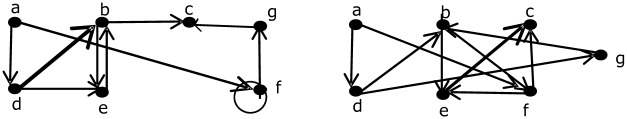
Sample graphs of anonymized network of Fig 2.

We evaluate our framework for two kinds of query:

- **Aggregate tabular query:** Similarly to queries on tabular data, we do not consider relationships between individuals. We only assume that each individual has some attributes (quasi-identifiers, sensitive attributes, in-degree and out-degree). Each query of this kind is a count query *Q* = *count*(*σ*_C_(*N*)), where we use only the *and* operator between simple conditions (attribute = value or attribute in {*value*_1_, *value*_2_,…, *value_k_*}) on those attributes in C. It can be transformed to Q* = *count*(*σ*_C_(*AT*_1_⨝*AT*_2_⨝*DT*⨝*ST*)) on released ASN. There is no foreign key between these tables, so lots of false tuples are generated by lossy join of those tables. Thus, we estimate the result of query Q on released ASN by $Q^{*}\;=\;\sum_{j\;=\;1}^{n^{'}}\left(\frac{sum\left(\pi_{count}(\sigma_{C_{AT1}\wedge \;Gid\;=\;j}\left({AT}_{1}\right))\right)}{\left|G_{j}\right|}*\frac{sum\left(\pi_{count}(\sigma_{C_{AT2}\wedge \;Gid\;=\;j}\left({AT}_{2}\right))\right)}{\left|G_{j}\right|}*\frac{count(\sigma_{c_{DT}\wedge \;Gid\;=\;j}\left(DT\right))}{\left|G_{j}\right|}*sum\left(\pi_{count}(\sigma_{C_{S}\wedge \;Gid\;=\;j}\left(ST\right))\right)\right)$, where we separate conditions related to each table (*C* = *C*_AT1_∧*C*_AT2_∧*C_DT_*∧*C_ST_*). Some of these conditions may be true if there is no condition related to attributes of its table in C condition, and *n*′ is the number of anonymized groups. We generate two kinds of queries: a) equality query, where all conditions have equality operator; b) range query, where all conditions except the condition on sensitive attribute are range conditions. We generate 100 random queries from each kind.
- **Graph topological properties:** The anonymized network data produced by ASN is analyzed in the same way as network generalization methods [6,4], whereby sample graphs are generated from the published data. So, we randomly generate 100 sample graphs based on released tables (DT and SVT). As mentioned before, $\prod_{j\;=\;1}^{n'}\left|VEC(G_{j})\right|$ graphs could be reconstructed. One member of *valid edges choices* set of each group is generated randomly to reconstruct each sample graph. These graphs show structural information of the network. We run topological queries on these graphs, and compute the average of each query response for all sample graphs. Fig 5 shows two sample graphs of the anonymized network from Fig 2. We compute information loss of the following measures for reconstructed graphs to evaluate preserved data utility of the proposed framework: diameter, shortest path length, clustering coefficient, closeness, betweenness, size of maximum strongly and weakly connected components.
- **The graph spectrum** has close relations with many graph characteristics and can provide global measures for some network properties. We consider the normalized eigenvector metric. The eigenvector is a non-zero vector *v* = {*v*_1_,…, *v_n_*}; when graph adjacency matrix *A*_n×n_ is multiplied by *v*, it yields a constant multiple of *v*. The normalized eigenvector equals $nv\;=\;\frac{1}{\sum_{i\;=\;1}^{n}v_{i}}v$.

**Fig 6:**
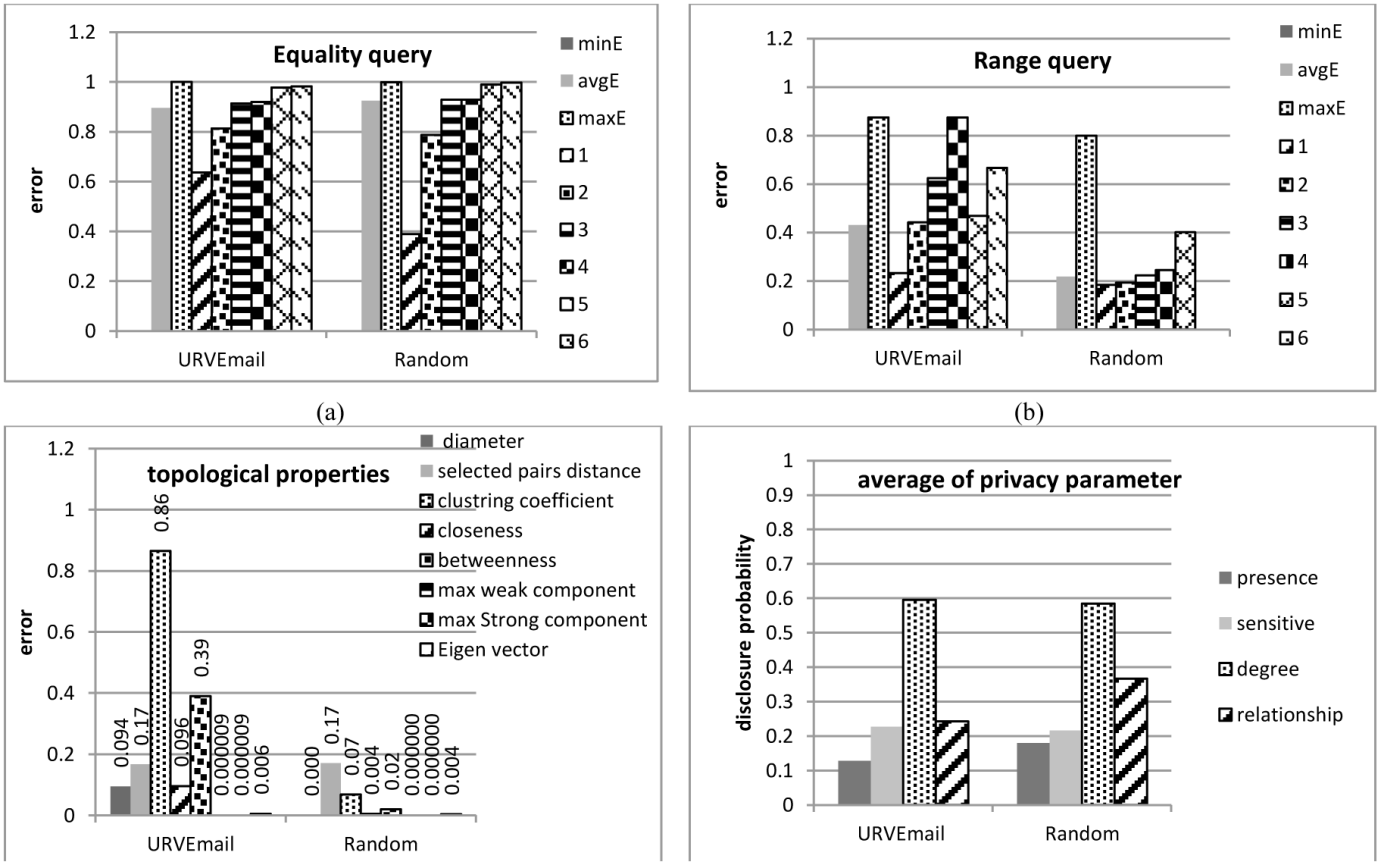
Information loss and privacy of released social network data.

Let *Q* be a query, and *Q*(*N*) and *Q*(N*) be accurate and approximate results, applying *Q* to the original network data *N* and the anonymized network data N*, respectively, based on the proposed framework. The relative error is $Error\;=\;\frac{\left|Q\left(N\right)-Q\left(N^{*}\right)\right|}{\left|Q\left(N\right)\right|}$. This relative error is computed for each aggregate tabular query. Less relative error shows less information loss and more data utility. Fig 6(a) and 6(b) show minimum, average and maximum error of total generated random equality and range queries in both network datasets. In each kind of query, the error of the Random network is less than that of the URVEmail network. In addition, for each network dataset, the relative error of the equality query is more than that of the range query. We define selectivity of one query as the number of individuals that satisfy all its conditions. Since the selectivity of equality query is less than that of range query, the result for the equality query is less. So, based on the Error formula, a small change in small actual value causes more relative error compared to large actual value. Fig 6(a) and 6(b) also illustrate average relative error with respect to the number of properties involved in conditions of the queries. As shown, an increased number of involved properties increases relative error due to decrease in query selectivity.

Fig 6(c) shows the average relative error in structural and spectrum properties of all sample-generated graphs from the released network data. To evaluate information loss in the shortest path lengths between individuals, we generate 100 random pairs with paths between them in the original graph, then compute the average relative error distance of each selected pair. Since closeness, betweenness, and clustering coefficient of some nodes in the original network may be zero, to avoid dividing by zero in Error, we use $ErrorDA\;=\;\frac{{\text{avg}}_{v\in V}\left|Q\left(v\right)-Q(v^{*})\right|}{{\text{avg}}_{v\in V}Q\left(v\right)}\;(v$* denotes the corresponding *v* in N*) for each reconstructed graph. As shown, our framework preserves all structural properties very well for the Random dataset, and for the URVEmail dataset at an acceptable level. In addition, degree distribution remains unchanged in the proposed framework.

In the Random dataset the range of in-degree and out-degree of all nodes is limited and degree distribution of nodes is relatively uniform. In this case, finding a grouping to satisfy degree and relationship privacy is easy. URVEmail has power-law degree distribution. In this dataset, more than half of the nodes have degree under 15; there are a few nodes with degree greater than 50. In this dataset, it is difficult to find grouping based on ASN such that all its groups satisfy relationship privacy. This situation causes group size to be increased, and the information loss for the URVEmail network is more than that for the Random dataset. It is necessary to mention that relative error near to 1 is not so undesirable when the actual value is near to 0, because even a small change in anonymized value causes a considerable increase in relative error.

Fig 6(d) presents the average privacy parameters of all network individuals, all of them are under their thresholds.

## Conclusion and Future Work

Anonymization is a good technique to preserve specified privacy in social-network data publishing. In this paper, we introduce a novel anonymization technique in terms of an ASN that considers four privacy constraints to prevent disclosure of presence, sensitive-attribute, degree and relationship. ASN breaks associations between properties of each individual by storing them in separate tables. This scheme preserves data utility of tabular, structural and spectrum properties at a satisfactory level. The main features of ASN are as follows: 1) some data, such as degree and sensitive attribute may belong to both private information and background knowledge; 2) different privacy thresholds are used for different kinds of private information depending on their disclosure importance; 3) the degree distribution of nodes, which has an important effect on topological properties, remains unchanged.

In ASN, the method of partitioning nodes within groups is strongly related to usage of published data (dependent on application) and has an important effect on data utility. An important area for future work is to develop an anonymization algorithm for ASN that considers some data utility terms in a desirability metric. It would also be possible to extend our privacy model to consider privacy constraints for relationship types and multiple sensitive attributes; thus, in the proposed anonymization technique, separate tables for each sensitive attribute that is the same as ST and one table for the type of output edges of each group with scheme (GID, Rtype, count) should be considered. In addition, we will consider different privacy levels [45] for different individuals in social-network data.

## Supporting Information

S1 Dataset The real dataset (URVEmail). (RAR)

S2 Dataset The synthetic dataset (Random). (RAR)
